# Perceived social support and depression among people living with HIV in China: roles of stigma and adherence self-efficacy

**DOI:** 10.1186/s12888-023-04997-1

**Published:** 2023-07-26

**Authors:** Pengfei Wang, Jianjing Xiong, Jinlei Zheng, Chengliang Chai, Ying Wang

**Affiliations:** 1 School of Public Health, Fudan University, No. 130, Dong’an Road, Shanghai, 200032 China; 2 NHC Key Laboratory of Health Technology Assessment, Fudan University, No. 130, Dong’an Road, Shanghai, 200032 China; 3 Jing’an District Center for Disease Control and Prevention, No. 195, Yonghe Road, Shanghai, 200072 China; 4 Zhejiang Provincial Center for Disease Control and Prevention, No. 3399, Binsheng Road, Hangzhou, 310021 Zhejiang China

**Keywords:** People living with HIV, Depression, Perceived social support, Stigma, Adherence self-efficacy

## Abstract

**Introduction:**

People living with HIV (PLHIV) are a high-risk group for depression. In particular, the prevalence and burden of depression is higher and more severe among PLHIV in developing and less-developed countries. There is evidence that perceived social support has a positive impact on reducing the occurrence of depression, and high stigma and low adherence self-efficacy are barriers to the effectiveness of social support for depressed PLHIV. However, how these risks affect the effect of social support on depression still needs further identification.

**Methods:**

Between 2017 and 2018, a total of 1139 Chinese PLHIV (74.36% male, mean age = 43.91 years) from three provinces (Shanghai, Zhejiang and Henan) in China were enrolled in the study. Data were analyzed by multiple regression, mediation model, and moderation model.

**Results:**

A total of 43.99% of PLHIV had mild to severe depression. There was a significant negative association between perceived social support and depression (B = -0.049, *P* < 0.05). Stigma and adherence self-efficacy played a chain mediating role (B = -0.058, 95% CI: -0.078 ~ 0.039) and a moderating role in the effect of perceived social support on depression (stigma: B = -0.003, *P* < 0.05; adherence self-efficacy: B = 0.004, *P* < 0.05).

**Conclusion:**

Stigma and adherence self-efficacy indirectly predicted depression, and perceived social support was more effective in reducing depression among PLHIV with high stigma or low adherence self-efficacy. Enhancing multiple social support resources for PLHIV may reduce their risk of depression. Moreover, the need for social support is greater for those with high stigma or low adherence self-efficacy.

## Introduction

People living with HIV (PLHIV) are at high risk for depression. Many studies have shown that most PLHIV experience higher levels of anxiety, depression, and frustration than the general public [1, 2]. PLHIV are more likely to develop psychiatric disorders, especially depression, due to social discrimination, physical discomfort, chronic illness, side effects of treatment, and neurobiological changes. The global prevalence of depression in PLHIV is 31%, which is higher in developing and less-developed countries than in developed countries [3]. PLHIV in China are facing a serious threat of depression. A review that included 20,635 Chinese PLHIV showed that the combined prevalence of depression was 50.8% among general PLHIV [4]. Depression may worsen existing disease states, complicate the course of the disease, increase mortality on PLHIV [5, 6], and place a heavy burden on health systems [7]. Therefore, identifying and exploring depression and its related mechanisms are essential to developing mental health interventions, which are becoming increasingly important to improve the mental and physical quality of life in PLHIV.

Perceived social support involves subjective evaluations of how people perceive support from friends, families, and others who can provide material and psychological support when they need help. The association between perceived social support and mental health is strong and well-established. Current studies have shown that, in PLHIV, depression can be attributed to low levels of perceived social support [8, 9]. However, limited studies have explored how and when social support affects depression in PLHIV.

HIV-related stigma is considered discrete and discriminatory against PLHIV. In the decades since the HIV epidemic, even though many efforts have been made, PLHIV still experience a higher level of stigma than populations with other chronic diseases. Studies showed that stigma is a risk factor for depression in PLHIV, is positively associated with depression [10, 11], and is negatively associated with healthy psychological states, such as well-being [12]. A growing body of research shows that social support can reduce HIV-related stigma [13, 14]. This suggests that there may be a pathway of social support to reduced depression through reduced stigma [15, 16]. One study suggested that clinicians may be recommended to consider the association between stigma and depression in their treatments and programs and that it is helpful to include social support as a protective factor, as it is a useful intervention for promoting mental health in PLHIV [17].

Higher treatment adherence self-efficacy indicates more psychological confidence and persistence in adhering to treatment [18]. Several studies have demonstrated that medication adherence is negatively associated with depression. For example, the risk of moderate-to-severe depressive symptoms was reported to be three times higher in patients who were not adherent to antiretroviral therapy (ART) than in those who were. Therefore, it was hypothesized that adherence self-efficacy would be negatively associated with depression in this study. This is supported by the results of two studies on viral suppression in women with HIV, which showed a relationship between depression and adherence self-efficacy, with severe depression being associated with lower adherence self-efficacy [19, 20]. In addition, stigma may have a negative impact on adherence self-efficacy. A study with 186 American PLHIV suggested that adherence self-efficacy mediates the association between reduced internalized stigma and viral suppression as well as treatment adherence [21]. This suggests a link between stigma and adherence self-efficacy as well. Social support is also associated with adherence self-efficacy, that is, receiving recent social support predicts higher adherence self-efficacy, and interventions targeting social support may improve adherence self-efficacy [22]. This suggests a possible role for stigma and adherence self-efficacy along the pathway of social support to depression. However, most current studies on adherence self-efficacy have focused on treatment adherence or physiological outcomes of PLHIV, and the role of adherence self-efficacy in mechanisms influencing psychological outcomes, such as depression, has received less attention [23].

This study had two aims: (1) to investigate the association between perceived social support, stigma, adherence self-efficacy, and depression; and (2) to determine whether stigma and adherence self-efficacy mediate or moderate the association between perceived social support and depression.

## Methods

### Study design and participants

A cross-sectional survey of ART adherence in PLHIV was conducted in Shanghai, Zhejiang Province, and Henan Province in China from November 2017 to November 2018. A multi-stage stratified random sampling method was used. PLHIV who received at least three months of ART in three provinces were divided according to the duration of ART into four strata and randomly selected from each stratum. The participants were recruited by the local Centerfor Disease Control and Prevention (CDC). Those who were recruited and provided written consent completed a face-to-face survey with structured questionnaires. Specifically, 281 participants from 14 communities of Jing’an District, Shanghai, 487 participants from nine districts of Zhoukou City, Henan Province, and 505 participants from six counties in six cities of Zhejiang Province completed the questionnaires. Of the total of 1273 participants who completed the survey, 1139 participants who provided complete information on the key variables were included in the final data analysis. The study was approved by the Review Board of Fudan University School of Public Health (IRB#2014–03-0497).

### Measures

#### Perceived social support

Perceived social support was measured using the Multidimensional Scale of Perceived Social Support (MSPSS) [24]. This scale contains 12 items that measure perceived social support from friends, family, and others. The items are scored on a seven-point Likert scale (1 = “very strongly disagree” to 7 = “very strongly agree”), with the total score ranging from 12 to 84. Higher scores indicate higher levels of perceived social support. MSPSS has been proved to be reliable and valid with an overall Cronbach’s α ranging from 0.84 to 0.92 [23]. For this study, the Cronbach's α for the MSPSS was 0.928, showing good reliability in our survey.

#### Depression

Depression was measured using the Beck Depression Inventory-II (BDI-II) [25]. This scale includes 21 items that reflect the presence and severity of the affective, cognitive, and somatic symptoms of depression. The items are scored on a four-point scale (0 = “symptoms absent,” 1 = “symptoms present,” 2 = “moderate symptoms,” and 3 = “severe symptoms”), with the total score ranging from 0 to 63. In our study, scores < 14 reflecting no depression, 14–19 reflecting mild depression, 20–28 reflecting moderate depression and 29–63 reflecting severe depression. The BDI-II showed good internal consistency as Cronbach’s α was 0.93 among PLHIV [26]. Cronbach’s α of the BDI-II was 0.923, showing good reliability in this study.

#### HIV-related stigma

HIV-related stigma was measured by the Berger HIV stigma scale (BHSS) [27], which includes 40 items on a four-point Likert scale (1 = “strongly disagree,” 2 = “disagree,” 3 = “agree,” and 4 = “strongly agree”). The total score ranges from 40 to 160. Higher scores suggest more severe stigma due to PLHIV. The BHSS appears to be a reliable and valid measure of HIV-related stigma with Cronbach's α more than 0.70 [28]. Cronbach’s α of the BHSS was 0.938 in this study, also showing good reliability.

#### HIV treatment adherence self-efficacy

HIV treatment adherence self-efficacy was measured using the Adherence Self-Efficacy Scale (HIV-ASES) [29]. The scale was developed to measure self-efficacy for adherence to HIV treatment plans, including nutrition, exercise, and taking HIV medications, such as ART. This reflects the confidence, integration, and perseverance in following, controlling, and managing treatment in PLHIV. The scale includes 12 items that are rated on a response scale from 0 (‘‘cannot do at all”) to 10 (‘‘completely certain can do’’). Higher scores indicate greater adherence self-efficacy. The Chinese version of the HIV-ASES has high reliability and validity, and its Cronbach’s α was 0.876 [30]. Cronbach’s α of the HIV-ASES was 0.951 in this study, showing good reliability.

### Statistical analysis

SPSS software (version 25.0) was used for data analysis. Categorical variables were presented as numbers and frequencies. Continuous variables were presented as means and standard deviations (SD). The associations between key variables (perceived social support, stigma, adherence self-efficacy, and depression) were analyzed using partial correlations after controlling for covariates (age, gender, education, employment, marriage, monthly income, smoking, drinking, and time since HIV diagnosis).

In statistics, a mediation model attempts to identify and explain the mechanism or process of the observed relationship between the predictor and outcome variables by including a third hypothetical variable, which is called a mediating variable. Mediation analysis is used to explore the potential mechanisms or processes by which one variable influences another variable through a mediating variable [31]. In the present study, stigma and adherence self-efficacy were used as mediating variables to explore their roles in the potential mechanisms between social support and depression. In statistical and regression analysis, moderation occurs when the relationship between two variables depends on a third variable, which is referred to as the moderating variable [31]. The moderating variable affects the direction and/or strength of the relationship between the predictor variable and outcome variable. This study also explored the moderating role of stigma and adherence self-efficacy between social support and depression, that is, it explored at what level of stigma and adherence self-efficacy the strength of the effect of social support on depression was greater.

For the mediating effect, Model 6 in PROCESS of SPSS was used to examine the chain-mediating effect of stigma and adherence self-efficacy between perceived social support and depression. For the moderating effect, Model 1 and Model 2 in PROCESS were used to test three moderated models, in which perceived social support served as the predictor variable and depression served as the outcome variable. In the first model, stigma served as a moderating variable. In the second model, adherence self-efficacy served as a moderating variable, and in the third model, stigma and adherence self-efficacy together served as moderating variables. The bootstrap 95% confidence interval (CI) was used to estimate the chain-mediating effect and moderating effect based on 5000 bootstrap samples. All analyses were controlled for the covariates.

## Results

### Participant characteristics

Table 1 shows the participants’ characteristics. A total of 1139 PLHIV were enrolled; their average age was 43.91 years (SD = 13.78, range from 14 to 84 years); and 74.36% were male. A total of 52.59% of participants had elementary or middle school education; 49.69% of the participants were employed or students, and the others were unemployed, retired, or otherwise. Further, 52.68% of participants were separated, divorced, or unmarried; 39.33% of participants had a monthly income of 0–1500 yuan. Regarding their lifestyle habits, 76.73% of the participants never smoked and 68.13% never drank alcohol. The average time since HIV diagnosis was 7.19 years (SD = 5.84). There were 56.01% of the participants who did not have depression, and 43.99% who had mild to severe depression.

**Table 1:** Descriptive statistics of the sociodemographic variables. (*N* = 1139)

	**N (%)**
**Age (years)**	
≤ 30	232 (20.37)
31 ~ 45	381 (33.45)
46 ~ 60	371 (32.57)
> 60	155 (13.61)
**Gender**	
Male	847 (74.36)
Female	292 (25.64)
**Education**	
Elementary or middle school	599 (52.59)
High School	172 (15.10)
College or university and above	368 (32.31)
**Employment**	
Employed/Students	566 (49.69)
Unemployed/Retired/Others	573 (50.31)
**Marital status**	
Married/Cohabitating	539 (47.32)
Separated/Divorced/Unmarried	600 (52.68)
**Monthly income (yuan)**	
0–1500	448 (39.33)
1501 ~ 3000	210 (18.44)
3001 ~ 4500	120 (10.54)
> 4500	361 (31.69)
**Smoking**	
Never	874 (76.73)
Sometimes	144 (12.64)
Often	121 (10.62)
**Drinking alcohol**	
Never	776 (68.13)
Sometimes	320 (28.09)
Often	43 (3.78)
**Time since HIV diagnosis (years)**	
< 3	311 (27.30)
3 ~ 5	300 (26.34)
6 ~ 8	144 (12.64)
> 8	384 (33.71)
**Depression (BDI-II score)**	
No	638 (56.01)
Mild	180 (15.80)
Moderate	211 (18.53)
Severe	110 (9.66)

### Correlation analysis and regressions of perceived social support, stigma, adherence self-efficacy, and depression

Table 2 shows partial correlations among key variables after controlling for all covariates. All key variables were significantly associated. Perceived social support and adherence self-efficacy were negatively associated with stigma (r = -0.162, *P* < 0.001; r = -0.123, *P* < 0.001) and depression (r = -0.126, *P* < 0.001; r = -0.296, *P* < 0.001). Perceived social support was negatively associated with adherence self-efficacy (r = 0.138, *P* < 0.001). Stigma was positively associated with depression (r = 0.237, *P* < 0.001).

**Table 2:** Partial correlations between social support, stigma, adherence self-efficacy and depression

	**Mean (SD)**	**Perceived social support**	**Stigma**	**Self-efficacy**	**Depression**
Perceived social support	59.09(12.20)	-			
Stigma	111.20(14.95)	-0.162***	-		
Self-efficacy	110.53(14.60)	0.138***	0.123***	-	
Depression	13.46(10.35)	-0.126***	0.237***	-0.296***	-

Correlations between key variables were analyzed using partial correlations analysis. All correlations were controlled for age, gender, education, employment, marital status, monthly income, smoking, drinking, and time since HIV diagnosis

^***^ *P* < 0.001

Table 3 shows the results of the regression analysis of key variables after controlling for the covariables. Perceived social support negatively predicted stigma (B = -0.210, *P* < 0.001). Perceived social support (B = 0.155, *P* < 0.001) and stigma (B = -0.102, *P* < 0.001) significantly predicted adherence self-efficacy. Perceived social support (B = -0.049, *P* < 0.05), stigma (B = 0.128, *P* < 0.001), and adherence self-efficacy (B = -0.175, *P* < 0.001) independently predicted depression.

**Table 3:** Regression analysis of the variables in the chain-mediating model

Outcome variable	Predictor variable	B	SE	t	*P*	LLCI	ULCI
**Stigma (Model 1)**	Perceived social support	-0.210	0.038	-5.528	< 0.001	-0.284	-0.135
**Adherence Self-efficacy (Model 2)**	Perceived social support	0.155	0.038	4.1081	< 0.001	0.080	0.229
	Stigma	-0.102	0.029	-3.481	< 0.001	-0.160	-0.045
**Depression (Model 3)**	Perceived social support	-0.049	0.024	-2.042	< 0.05	-0.096	-0.002
	Stigma	0.128	0.019	6.903	< 0.001	0.091	0.164
	Adherence self-efficacy	-0.175	0.019	-9.346	< 0.001	-0.212	-0.138

All regression analyses were controlled for age, gender, education, employment, marital status, monthly income, smoking, drinking, and time since HIV diagnosis

Model 1 summary: *R* = 0.255; R-sq = 0.065; *F* = 7.8739; *P* < 0.001

Model 2 summary: *R* = 0.225; R-sq = 0.051; *F* = 5.461; *P* < 0.001

Model 3 summary: *R* = 0.504; R-sq = 0.254; *F* = 31.986; *P* < 0.001

### The mediating roles of stigma and adherence self-efficacy in the effect of perceived social support on depression

**Fig. 1:**
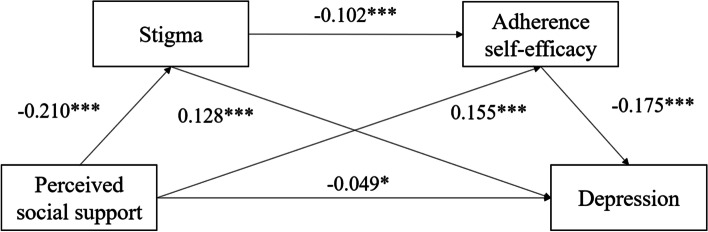
The specific path of the associations between perceived social support and depression (mediating effect). *P < 0.05, ***P < 0.001

Table 4 and Fig. 1 show the results of the mediating effects of stigma and adherence self-efficacy and the path of the model. The results show that the mediation effect (B = -0.058, 95% CI: -0.078 ~ -0.039), direct effect (B = -0.049, 95% CI: -0.096 ~ -0.002), indirect effect 1 (B = -0.027, 95% CI: -0.043 ~ -0.014), indirect effect 2 (B = -0.027, 95% CI: -0.043 ~ -0.013), and indirect effect 3 (B = -0.004, 95% CI: -0.007 ~ -0.010) were all significant.

**Table 4:** Analysis of the mediating effect of stigma and adherence self-efficacy

	**Effect size**	**SE**	**Boot LLCI**	**Boot ULCI**	**Relative mediation effect**
Direct effect	-0.049	0.024	-0.096	-0.002	
Indirect effect 1	-0.027	0.007	-0.043	-0.014	25.23%
Indirect effect 2	-0.027	0.008	-0.043	-0.013	25.23%
Indirect effect 3	-0.004	0.002	-0.007	-0.010	3.74%
Total mediation effect	-0.058	0.010	-0.078	-0.039	54.20%

Indirect effect 1: Perceived social support → Stigma → Depression

Indirect effect 2: Perceived social support → Adherence self-efficacy → Depression

Indirect effect 3: Perceived social support → Stigma → Adherence self-efficacy → Depression

### The moderating roles of stigma and adherence self-efficacy in the effect of perceived social support on depression

**Fig. 2:**
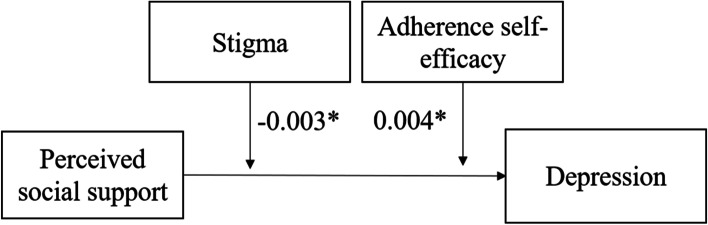
The specific paths of the associations between perceived social support and depression (moderating effect). *P < 0.05

Figure 2 shows the paths of the three moderation effect models. Table 5 shows the moderating roles of stigma and adherence self-efficacy. The results demonstrated that stigma (B = -0.003, *P* < 0.05), adherence self-efficacy (B = 0.003, *P* < 0.05), and the joint effect of stigma (B = -0.003, *P* < 0.05) and adherence self-efficacy (B = 0.004, *P* < 0.05) had moderating effects on the association between perceived social support and depression.

**Table 5:** Analysis of the moderating effect of stigma and adherence self-efficacy

	**Model 1** **B (SE)**	**Model 2** **B (SE)**	**Model 3** **B (SE)**
Perceived social support	-0.074 (0.025) **	-0.074 (0.024) **	-0.047 (0.024) *
Stigma	0.152 (0.019) ***	-	0.137 (0.019) ***
Adherence self-efficacy	-	-0.180 (0.019) ***	-0.165 (0.019) ***
Stigma * perceived social support	-0.003 (0.001) *	-	-0.003 (0.001) *
Adherence self-efficacy * perceived social support	-	0.003 (0.002) *	0.004 (0.002) *

*SE* Standard Error. Model 1 presents the moderating effect of perceived social support on depression through stigma. Model 2 presents the moderating effect of perceived social support on depression through adherence self-efficacy. Model 3 presents the moderating effect of perceived social support on depression through stigma and adherence self-efficacy

Model 1 summary: *R* = 0.446; R-sq = 0.199; *F* = 23.331; *P* < 0.001

Model 2 summary: *R* = 0.475; R-sq = 0.225; *F* = 27.293; *P* < 0.001

Model 3 summary: *R* = 0.511; R-sq = 0.262; *F* = 28.427; *P* < 0.001

^*^P < 0.05

^**^P < 0.01

^***^P < 0.001

**Fig. 3:**
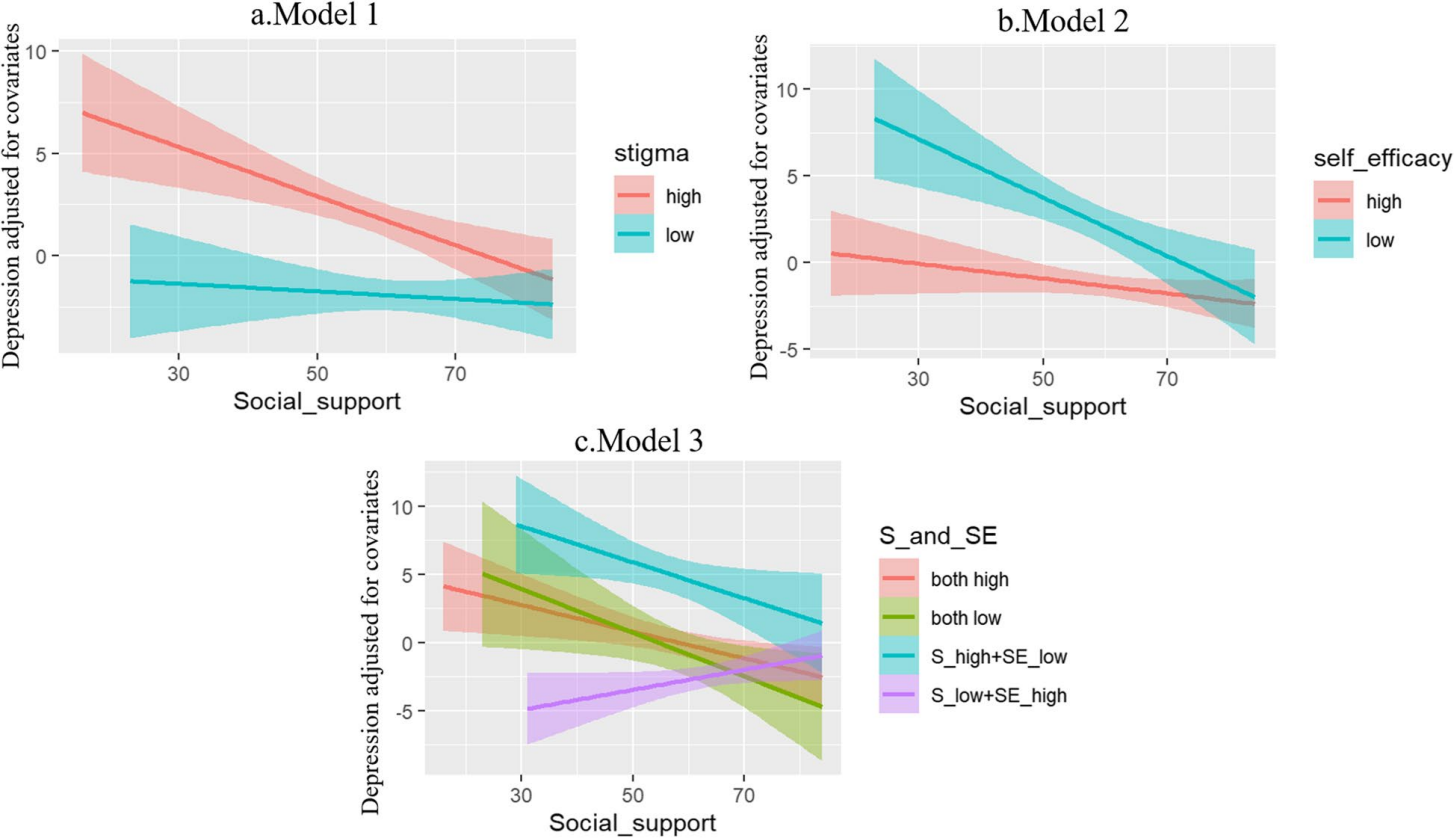
The moderation effects of stigma and adherence self-efficacy in the effect of perceived social support on depression. Note: *S* Stigma, *SE* Self-efficacy

Figure 3 shows that high stigma levels increased the association between perceived social support and depression. The difference in depression scores between the high-stigma group and the low-stigma group became smaller as perceived social support scores increased. Low adherence self-efficacy increased the association between perceived social support and depression. Adherence self-efficacy moderated the association between perceived social support and depression, and as social support increased, the difference in depression scores between the “high adherence self-efficacy” group and the “low adherence self-efficacy” group became smaller. The association between perceived social support and depression was moderated by the “high stigma and low self-efficacy” group as well as the “low stigma and high self-efficacy” group, but the results were similar for the “both high” group and “both low” group. As the perceived social support scores increased, the difference in depression scores between the “high stigma and low self-efficacy” group and the “low stigma and high self-efficacy” group became smaller. In the case of the “high stigma and low self-efficacy” group, depression scores decreased as perceived social support scores increased, whereas in the case of the “low stigma and high self-efficacy” group, depression scores increased as perceived social support increased. The “both high” and “both low” groups had moderating effects compared to the “high stigma and low self-efficacy” group, and both had lower depression scores than the “high stigma and low self-efficacy” group, with depression decreasing as perceived social support increased.

## Discussion

The aim of this study was to explore the potential psychological mechanisms underlying the association between perceived social support and depression, including the mediating and moderating roles of HIV-related stigma and adherence self-efficacy. Participants were PLHIV in Shanghai, Zhejiang, and Henan provinces in China. A total of 43.99% of PLHIV in central and east China in our study were reported to have mild to severe depression, which is similar to that obtained from the studies of depression of PLHIV in China measured with the same scale and cut-off values [32, 33]. While the significant effects of perceived social support on depression are supported by several studies, the underlying mechanisms of this relationship in PLHIV remain to be discussed. The present study broadened the focus of depression research among PLHIV and presented a more complex hypothesis of how perceived social support affects depression. This study revealed a negative association between perceived social support and depression in PLHIV, which is consistent with previous studies. More importantly, the positive results of this study suggest important roles for two important indicators of psychological status in PLHIV—stigma and adherence self-efficacy—in the reduction of depression in PLHIV by high levels of perceived social support. According to current studies, PLHIV with less perceived social support experience more severe depression because of higher stigma and lower adherence self-efficacy. At the same time, perceived social support had a stronger effect on depression in PLHIV with high stigma or low adherence self-efficacy.

This study suggests that perceived social support affects depression in PLHIV by reducing stigma, which adds quantitative evidence to how social support affects depression. PLHIV who feel more social support will have a greater sense of self-worth, strength, and courage, and feel included and cared for by others during infection and treatment, all of which contribute to a reduction in HIV-related stigma and thus a reduced risk of depression [34, 35].

This study explored, for the first time, the mediating role of adherence self-efficacy on the relationship between perceived social support and depression among PLHIV. In contrast to previous studies that linked adherence self-efficacy to ART treatment adherence, this study found a direct link from adherence self-efficacy to depression as well. The association between social support and adherence self-efficacy has been demonstrated [36, 37]: the positive effects of social support can be extended to specific constructs of adherence self-efficacy; interventions targeting social support processes can improve adherence self-efficacy [22]; and increased adherence self-efficacy is often accompanied by increased treatment adherence. This study proposes a novel pathway to improve adherence self-efficacy in PLHIV through social support that may not only positively influence treatment adherence but may also reduce depression levels.

Our findings support our hypothesis that stigma and adherence self-efficacy have a chain mediating effect on the relationship between perceived social support and depression in PLHIV. The findings suggest that HIV-related stigma is reduced when a high level of social support is available and that reduced stigma may be associated with increased confidence and perseverance in treatment, which has an impact on depression. Several psychosocial variables have been mentioned to mediate the relationship between social support and adherence self-efficacy, one of which is HIV-related stigma. Specifically, the association between social support and adherence self-efficacy was stronger in individuals with lower levels of stigma [22]. Perceived social support helps PLHIV overcome fear and stigma, resulting in confidence and perseverance to adhere to treatment, leading to a better psychological state. Thus, the chain from stigma to treatment adherence self-efficacy is an important bridge from perceived social support to depression in PLHIV.

Additionally, this study found that stigma and adherence self-efficacy played moderating roles in the relationship between perceived social support and depression. The effect of perceived social support on depression differed for PLHIV with different stigma or adherence self-efficacy levels. Perceived social support had a greater impact on depression when stigma was high or adherence self-efficacy was low, suggesting that such populations benefit more from social support, and implementing interventions targeting social support can lead to better mental health improvement outcomes. Interestingly, we found that higher social support did not reduce depression levels when stigma was low and adherence self-efficacy was high, suggesting that the onset of depression in this type of PLHIV may not be related to the level of perceived social support.

Taken together, these results have practical implications: (1) it is important to focus on stigma and adherence self-efficacy when implementing social support interventions, and (2) it is critical to recognize the urgency of social support for depressed PLHIV with high stigma or low adherence self-efficacy. From the results of this study, we can infer that social support interventions aimed at reducing stigma and increasing adherence self-efficacy are necessary. Interventions include using support groups, involving people in available community support programs, and helping them establish or rebuild supportive relationships with friends, family, and others who may be unique to PLHIV. These interventions can provide psychological counseling and health education for treatment adherence. The results of this study provide a new perspective to explore the association between perceived social support and depression.

To the best of our knowledge, this is the first study to explore the roles of stigma and adherence self-efficacy in the effects of perceived social support on depression, including chain-mediating roles and moderating roles. These findings suggest a novel psychological mechanism underlying depression in PLHIV.

### Limitations

There are several limitations to this study. First, the cross-sectional nature of this study precludes causal inferences. Second, self-reported findings may not be sufficiently objective. Social desirability, respondent bias, and recall bias might influence the results, such as self-reported lifestyle habits (smoking, drinking alcohol), diagnostic time, and responses in key variable scales in this study. Although this study suggests that stigma and adherence self-efficacy can be considered potential psychological mechanisms underlying the relationship between social support and depression, future studies should explore the role of other variables in this relationship.

## Conclusion

PLHIV are at higher risk of developing depression. Understanding how to reduce their depression is the focus of academic research. Our findings suggest that there is an association between perceived social support and depression, while HIV-related stigma and adherence self-efficacy can indirectly predict depression. Perceived social support has a stronger effect on depression in PLHIV who have high stigma or poor adherence self-efficacy. Therefore, stigma and adherence self-efficacy are two important variables in the association between perceived social support and depression. Our findings provide a reference and new perspective for the exploration of psychological mechanisms that may help guide new interventions for depression in PLHIV.

## Data Availability

Data supporting the results of this study are available from the author (YW) upon reasonable request and approval.
